# Comparative Evaluation of Obturating Techniques in Primary Teeth: An *in vivo* Study

**DOI:** 10.5005/jp-journals-10005-1309

**Published:** 2015-09-11

**Authors:** Kriti Vashista, Meera Sandhu, Vinod Sachdev

**Affiliations:** Postgraduate Student, Department of Pedodontics and Preventive Dentistry ITSCDSR, Ghaziabad, Uttar Pradesh, India; Professor, Department of Pedodontics and Preventive Dentistry ITSCDSR, Ghaziabad, Uttar Pradesh, India; Professor, Department of Pedodontics and Preventive Dentistry ITSCDSR, Ghaziabad, Uttar Pradesh, India

**Keywords:** Lentulospirals, Obturation, Pressure syringe, Primary teeth.

## Abstract

**Aim:** The present study was undertaken to compare two methods of obturation in primary teeth by using lentulospirals and pressure syringe, radiographically.

**Materials and methods:** Sixty teeth in subjects with mean age of 5.88 ± 1.58 years were obturated randomly using two different obturating techniques, i.e. group I: Thirty teeth obturated with pressure syringe, and group II: Thirty teeth obturated with lentulospiral. Quality of obturation and presence or absence of voids were assessed by taking radiographs after obturation was done using both the techniques. Results of quality of obturation were statistically analyzed using Chi-square test and Mann-Whitney’s test, whereas voids were analyzed using Chi-square test.

**Results:** No statistically significant difference between the quality of obturation using pressure syringe or lentulospiral (p > 0.05) was observed. However, significantly higher number of voids were found for lentulospiral technique as compared to pressure syringe (p < 0.01).

**Conclusion:** Both the techniques were found to be equally efficient statistically, though lentulospiral produced more voids.

**How to cite this article:** Vashista K, Sandhu M, Sachdev V. Comparative Evaluation of Obturating Techniques in Primary Teeth: An *in vivo* Study. Int J Clin Pediatr Dent 2015;8(3): 176-180.

## INTRODUCTION

Dentistry has evolved in an extremely refined and technologically developed profession. Earlier, the only rationale for pulpal treatment was to relieve pain. Currently, as the field of dentistry advances and has seen many scientific improvements, endodontics has an expanded role as it relates to preservation of healthy dental pulp. As primary teeth are the best space maintainers, preservation of intact primary dentition until eruption of the permanent successors is very important in maintaining integrity of the arch form.

Successful endodontic therapy needs preparation of an aseptic root canal and sealing of the root canal system. The ideal biomechanical endodontic treatment for the root canals of primary teeth is hard to achieve due to their fenestrated and tortuous nature.^1^ Therefore, major continued research is ongoing in the area of finding obturating materials and techniques, to suit the specific features of primary teeth.

The methods selected by the practitioners to fill the pulpectomized canals of primary teeth are numerous and varied. The obturation materials can be carried to the pulp chamber and canals by a lentulospiral, can be placed in bulk and pushed into the canals with an endodontic plug-ger or with a cotton pellet, and they can also be applied by using an endodontic pressure syringe.^2^ The other most common techniques for the delivery of obturating material to the apex of pulpectomized primary teeth include using amalgam pluggers , mechanical syringe, jiffy tube, tuberculin syringe.^3^

Many investigations have been carried out to evaluate and compare the success rate of different root canal filling materials and various obturating techniques for primary teeth. Previous *in vitro* investigations of methods of obturation in primary teeth showed good performance of the lentulospiral over other techniques.^3-5^

*In vitro* evaluation of root canal obturation methods in primary teeth have reported superiority of the lentu-lospiral mounted in a slow-speed handpiece in filling straight and curved root canals of primary teeth.^2^ Clinical evaluation of the lentulospiral and pressure syringe in obturation of root canals of primary teeth, however, has not yet been much investigated.^2^ We hypothesized that there be a significant difference in quality of obturation by two different obturation methods (pressure syringe and lentulospiral) in primary teeth. Null hypothesis to be tested was that there will not be a significant difference on quality of obturation by the two different obturation methods (pressure syringe and lentulospiral) in primary teeth.

Therefore, the present study was undertaken to compare two methods of obturation in primary teeth by using lentulospirals and pressure syringe, radiographically.

## MATERIALS AND METHODS

The present study was conducted in department of pedodontics and preventive dentistry at ITS center for dental studies and research, Muradnagar, Ghaziabad. Evaluation of the study was done by the ethical committee of the institute and ethical committee approval was taken prior to the study. Informed consent was taken from all the parents after explaining them the entire procedure in detail, before starting the treatment.

*Sample size estimation:* The sample size was estimated by using the given formula

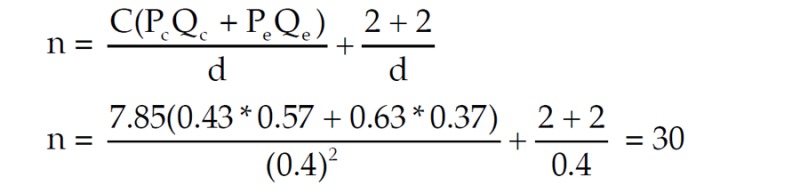



Based on the inclusion and exclusion criteria, 60 patients indicated for pulpectomy were selected in the study. Pulpectomy was performed in all teeth indicated for pulp therapy. Based on technique of obturation, patients were divided into two groups.

*Group I:* Thirty teeth obturated with pressure syringe.

*Group II:* Thirty teeth obturated with lentulospiral technique.

Children with the history of spontaneous pain, radiographs showing interradicular or periapical radiolucency, evidence of radicular pathologic lesion with caries involvement, clinically nonvital tooth with pus discharge, or continuous bleeding after amputation of coronal pulp tissue were included in our study. Nonrestorable tooth, tooth with pathological lesion extending to the successor tooth germ, tooth with evidence of external and internal root resorption were excluded from the study.

## CLINICAL PROCEDU

A standard preoperative radiograph was taken using conventional bisecting angle technique. Access to the pulp was obtained by round bur and barbed broach was used to remove it. The working length of the canal was established 1 mm short of radiographic apex. Biome-chanical preparation of root canal was done and the canal was irrigated using saline and dried using paper points. Obturation of the tooth was then done using either len-tulospiral technique or pressure syringe technique, and the teeth were divided in groups I and II, respectively.

### Obturation with Lentulospiral

A fine lentulospiral instrument was measured to 1 mm short of the predetermined canal length. The mixing ratio of zinc oxide and eugenol was two scoops of powder and two drops of liquid. The lentulospiral was dipped into the mixture, then introduced into the canal to its predetermined length and rotated into the canal. Additional amounts of paste were gradually introduced until the canal was filled. A radiograph was then taken for evaluation.

### Obturation with Pressure Syringe

A 22-gauge pressure syringe needle was selected and pre-fitted in the canal, with the length of the needle equaling 2 mm short of the predetermined canal length. The needle was placed in the prepared root canal to its previously observed depth. During continued filling of the canal with additional paste, the needle was withdrawn slightly to break contact with the side walls of the canals. This was followed by a radiograph for evaluation of obturation.

### Assessment of Obturation Techniques

The comparison among the two techniques was determined radiographically by evaluating quality of obturation and voids in the obturated canals, based on the following criteria given by Coll and Sadrian (1996)^6^:

- *Under filling (Score 1):* All the canals were filled more than 2 mm short of the apex.
- *Optimal filling (Score 2):* One or more of the canals having obturating material ending at the radiographic apex or upto 2 mm short of the apex.
- *Over filling (Score 3):* Any canal showing obturating material extending beyond the radiographic apex.
- *Voids:* Obturated canals showing voids (presence/ absence).

All the pulpectomies were completed and postoperative radiograph were taken immediately after the procedure. Assessment of radiographs were done by two different examiners who were unaware of the technique used for the obturation. Inter-examiner reliability test was performed and was found to be good.

## STATISTICAL ANALYSIS

Data collected were statistically analyzed using SPSS 18 software. Chi-square test and Mann-Whitney U test was applied to compare the obturating techniques. Filling of the obturated canals were evaluated using Chi-square tests (Tables 1 to 5), whereas voids were analyzed using Chi-square test (Table 2). Filling of the obturated canals were also evaluated using Mann-Whitney U test (Table 6).

## RESULTS

Sixty healthy children (43 males, 17 females) in the age group of 3 to 8 years with a mean age of 5.88 ± 1.58 years participated in our study. In total, 13 anterior teeth and 47 posterior teeth were obturated. The frequency distribution of 60 subjects with their age as 3 years was 6.7%, 4 was 15%, 5 was 23.3%, 6 was 15%, 7 was 18.3%, and 8 was 21.7%. On comparing the two techniques using Mann-Whitney’s test, no significant difference was found in the quality of obturation (p > 0.05) (Table 6). The difference in quality of obturation between anterior and posterior, maxillary and mandibular arches, and amongst gender was not found to be significant (p > 0.05) (Tables 3 to 5). However, significantly higher number of voids were found for lentulospiral technique as compared to pressure syringe (p < 0.01) (Table 2).

**Table 1:** **Table 1:** Comparison of quality of root canal filling with different obturation technique

		*Technique of obturation*					
*Quality of root* *canal filling*		*Pressure syringe* * N = 30*		*Lentulospiral* * N = 30*		*p-value*	
Underfilled		6 (20.0%)		5 (16.7%)		0.261	
Optimally filled		13 (43.3%)		19 (63.3%)		NS	
Overfilled		11 (36.7%)		6 (20.0%)			

p > 0.05

**Table 2:** **Table 2:** The frequency distribution of number of subjects with their obturation techniques and voids

		*Voids*					
*Technique of* * obturation*		*Present*		*Absent*		*p-value*	
Pressure syringe		7 (23.3%)		23 (76.6%)		0.002*	
Lentulospiral		19 (63.3%)		11 (36.6%)			

χ^2^ = 9.774, *p < 0.05

**Table 3:** **Table 3:** Frequency distribution of number of subjects on the basis of their gender by two obturation fill

				*Quality of root canal filling*							
*Gender*		*Technique of obturation*		*Underfilled*		*Optimum filled*		*Overfilled*		*p-value*	
Male		Pressure syringe		3 (13.6%)		11 (50%)		8 (36.4%)		0.185	
		Lentulospiral		2 (9.5%)		16 (76.2%)		3 (14.3%)		NS	
Female		Pressure syringe		3 (37.5%)		2 (25%)		3 (37.5%)		0.932	
		Lentulospiral		3 (33.3%)		3 (33.3%)		3 (33.3%)		NS	

**Table 4:** **Table 4:** Frequency distribution of number of subjects on the basis of their tooth position by two obturation techniques

		*Technique of obturation*												
		*Pressure syringe*							*Lentulospiral*					
*Type of obturated canals*		*Anterior teeth*		*Posterior teeth*		*p-value*			*Anterior teeth*		*Posterior teeth*		*p-value*	
Underfilled		0 (0%)		6 (20%)		0.079			1 (3.3%)		4 (13.3%)			
Optimum filled		5 (16.6%)		8 (26.6%)					6 (20%)		13 (43.3%)		0.275	
Overfilled		1 (3.3%)		10 (33.3%)					0 (0%)		6 (20%)		NS	

p > 0.05

**Table 5:** **Table 5:** Comparison of quality of root canal filling with different obturation techniques on the basis of arch

		*Technique of obturation*												
		*Pressure syringe*							*Lentulospiral*					
*Type of obturated canals*		*Maxillary arch*		*Mandibular arch*		*p-value*			*Maxillary arch*		*Mandibular arch*		*p-value*	
Underfilled		0 (0%)		6 (20%)		0.161			1 (3.3%)		4 (13.3%)			
Optimum filled		5 (16.6%)		8 (26.6%)					6 (20%)		13 (43.3%)		0.275 NS	
Overfilled		2 (6.6%)		9 (30%)					0 (0%)		6 (20%)			

p > 0.05

**Table 6:** **Table 6:** Mean rank scores of subjects in different obturation techniques

*Type of obturation*		*N*		*Mean rank*		*Mann-Whitney U*		*p-value*	
Pressure syringe		30		32.18		399.500		0.410	
Lentulospiral		30		28.82				NS	

p > 0.05

## DISCUSSION

On comparing both the obturation techniques, i.e. len-tulospiral and pressure syringe, no significant difference was found in the quality of obturation (p > 0.05), Since our results suggested that p > 0.05 (Table 1), so we accept null hypothesis, which depicted that both pressure syringe were equally effective for primary teeth obturation. In both the techniques, optimal filling was achieved in maximum number of root canals.

**Fig. 1:**
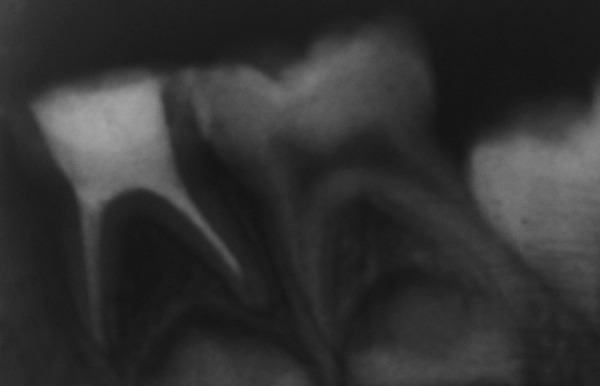
Underfilled root canal

**Fig. 2:**
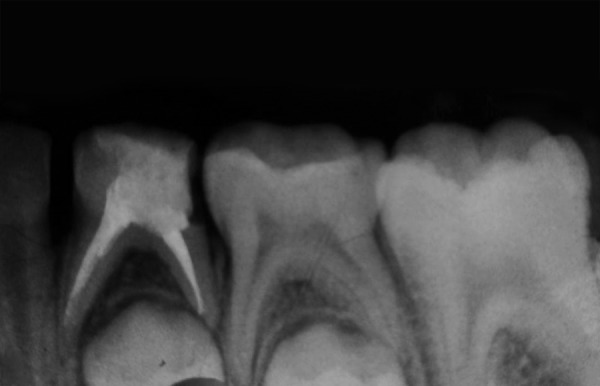
Optimum filled root canal

**Fig. 3:**
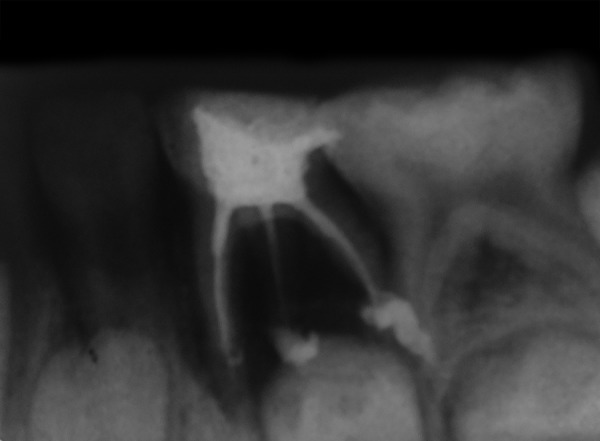
Overfilled root canal

The results of the present study showed that in group I, where the root canals were filled using pressure syringe technique, 43% of canals were optimally filled (Fig. 1); which shows one or more of the canals having obturating material ending at the radiographic apex or upto 2 mm short of the apex. A total of 20% of canals were under filled (Fig. 2) (2 mm short of the apex) and 36.7% of canals were overfilled, where obturating material extending beyond the apex. In the present study, more number of overfilled canals (Fig. 3) were observed with pressure syringe than under filled canals. This might be due to excessive pressure placed while placing the material into the canal, when the quarter turn of the screw was made. In the various studies, pressure syringe was proven to as a better technique for obturation when compared with incremental filling technique, lentulospiral technique.^5,6^

In group II, where handheld lentulospiral was used for root canal obturation, it was observed that 63% of the canals were optimally filled, 20% were overfilled and 16.7% were underfilled. Similar results were given by Sigurdsson et al, who compared sealer placement technique by endodontic file, syringe and lentulo drill; and found that lentulo drill presented best results, filling the entire working length.^7^ We also found that teeth obturated with lentulospiral showed less extrusion, which can be explained by the fact that the material is inserted in the canal by counter clockwise rotation of lentulospiral at predetermined length of the canal, rather than being pushed by pressure. These results are also well in correlation with Dandashi et al, who showed similar results, i.e. when they compared lentulospiral, pressure syringe and incremental technique, they too found less extrusion by incremental and lentulo technique.^5^

It can be concluded that lentulospiral gave better filling quality than pressure syringe although results were statistically non-significant. Our results are also in agreement with Greenber and Lee, which compared the lentulospiral and pressure syringe techniques, showing lentulospiral was superior than pressure syringe though the difference was not statistically significant.^5^

**Fig. 4:**
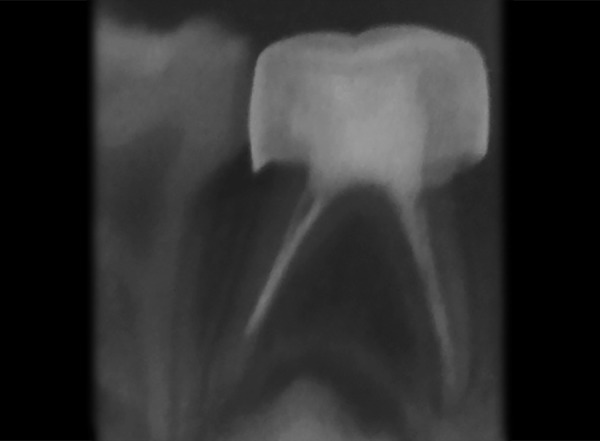
Underfilled root canal with void

When both the techniques were compared in terms of voids, we rejected the null hypothesis as it was observed that more uniform and dense root canal fill was seen when obturation was done with pressure syringe (Fig. 4). The possible reason could be the hub of the pressure syringe, being small and slender the thin needle provided a better reach till middle one-third of the canals, hence causing lesser voids. Pressure syringe gave better quality of root canal filling as compared to lentulospiral and the results are found to be statistically significant (p < 0.05) (Table 2). These findings are similar to those of Grover et al, who found that the endodontic pressure syringe was better than lentulospiral in controlling voids, as lentulospiral had the highest percentage of voids in canals (anterior 100%, posterior 75%) as compared to pressure syringe (anterior 12.5%, posterior 20%).^8^ Guelmann also found more voids with lentulospiral when compared with NaviTip.^10^ In another study by Dandashi, voids were also frequently observed, with the pressure syringe resulting.^5^

In the present study, assessment of the voids was done radiographically (intraoral periapical radiographs), which gave two dimensional view only, so it was not possible to find exact measurement and location of all the voids present; this can be a drawback of our study. Though in a study by Dandashi, voids were measured with the help of anterior/posterior and lateral radiographs; however, since it was an *in vitro* study, multiple views were taken.^5^ In our clinical study multiple views were not possible.

In various studies, the outcome of pulp therapy was also statistically compared between tooth types, such as maxillary and mandibular teeth, anterior and posterior, single rooted and multirooted teeth. Ng et al found statistically significant differences in success rates between tooth types. However, in our study we found that both the techniques gave similar results without any statistical significance (p > 0.05). It was observed in our study that the results of quality of obturation are independent of the arch in which the tooth is present.

Aylard and Johnson found that endodontic pressure syringe was superior for filling straight canals and lentulospiral was better for filling curved canals.^3^

Clinically, we found anterior canals were straight, so it was easy for the operator to obturate with both lentulospiral and pressure syringe; whereas, in posterior teeth it was easy to work with lentulospiral because of their flexibility.

Based upon the radiographic assessment, it was observed that both the techniques gave maximum number of optimal obturations. We found that while pressure syringe gave a compact filling, it was time consuming. On the other hand, lentulospiral was easy to use but the quality of root canal obturation was compromised due to more number of voids as compared to pressure syringe.

Hence, we conclude that there was no statistically significant difference between the use of pressure syringe or lentulospiral on the quality of root canal filling. Both the techniques gave maximum optimal obturations.
